# Outcome of Follow-Up Care Frequency on the Glycemic Control of Diabetic Patients in Qassim, Saudi Arabia

**DOI:** 10.7759/cureus.87842

**Published:** 2025-07-13

**Authors:** Omar M Aldhabbah, Saulat Jahan

**Affiliations:** 1 Family Medicine, Family Medicine Academy, Buraydah, SAU; 2 Public Health, Family Medicine Academy, Qassim Health Cluster, Buraydah, SAU

**Keywords:** cholesterol, electronic health records, follow up, glycated hemoglobin, glycemic control, hospital, retrospective study, saudi arabia

## Abstract

Aims

The aim of this study was to investigate the outcome of follow-up care frequencies on the glycemic control of diabetic patients in Qassim, Saudi Arabia.

Methods

A retrospective review of the electronic medical records of diabetic patients registered during the period August 2022 to July 2024 at King Fahad Specialist Hospital (KFSH) in Qassim was conducted. The association between follow-up care and various metabolic outcomes was determined by applying the chi-square test and Fisher's exact test. SPSS software version 22.0 (IBM Corp., Armonk, NY, US) was used for data analysis.

Results

The study included 286 patients with diabetes; 75.2% of them had glycemic control issues (HbA1c ≥ 7%), 56.3% of patients saw a doctor just once during the study period, and only 11.9% of patients received routine follow-up. Glycemic control and follow-up frequency were found to be significantly associated (χ² = 15.892, p < 0.001), with patients who received regular follow-up showing better results. Furthermore, poor glycemic control was significantly associated with low high-density lipoprotein (HDL) cholesterol (p = 0.015), but no significant associations were found with other lipid parameters.

Conclusions

In diabetic patients, better glycemic control has a direct relationship with regular follow-up care. These results emphasize the importance of organized, ongoing care in maximizing diabetes management as well as the inclusion of follow-up frequency into regular clinical practice.

## Introduction

Diabetes mellitus (DM) is becoming more prevalent worldwide. Diabetes is a chronic illness that arises from insufficient insulin production by the pancreas or the body's inability to utilize the insulin that is produced. Uncontrolled diabetes frequently results in hyperglycemia, also known as elevated blood glucose or elevated blood sugar, which, over time, seriously damages numerous organs and tissues, including the blood vessels and nerve cells [1]. The International Diabetes Federation reports that 537 million adults aged between 20 and 79 years have diabetes. By 2030, this figure is expected to increase to 643 million, and by 2045, it is expected to reach 783 million [2]. Diabetes increases the likelihood of having various health problems such as cardiovascular disease, renal disease, and retinopathy. However, people with diabetes who receive high-quality medical care with continuity experience fewer complications [3,4]. Even though many patients do not receive optimal-quality care and good results are rarely obtained, preventive medical care has the potential to improve health outcomes. A significant number of patients do not receive regular care, resulting in diabetic complications [5].

In primary care, regular follow-up care has been linked to lower rates of hospitalizations, reduced Emergency Room (ER) visits, increased patient satisfaction, and significantly lower healthcare expenditures [6]. Despite the importance of continuity of care, there are some limitations to accessing the services in some countries and difficulties in coordinating primary and secondary care. Longer doctor-patient relationships are substantially linked to lower mortality and fewer acute hospital admissions. The fact that continuity and these results have a dose-response connection suggests that these relationships are crucial [7]. Numerous studies looked into the connection between metabolic outcomes and follow-up frequency in type 2 DM (T2DM) patients. These findings led us to look more closely at the relationship between glycemic control and follow-up frequency [8]. Also, many studies have shown that continuous care plays a crucial role in improving metabolic control and reducing complications in people with diabetes [9,10]. However, there remains a gap in research that focuses on how this relationship applies in typical clinical settings. This study was conducted to investigate the impact of follow-up care on the glycemic control of diabetic patients. Furthermore, lipid profile, renal function, and liver function parameters were compared between T2DM patients with good glycemic control (HbA1c >7) and those with poor glycemic control (HbA1c <7).

## Materials and methods

Study design and setting

This is a retrospective record review of patients with diabetes who received follow-up care and were registered in King Fahad Specialist Hospital (KFSH), Buraidah City, Qassim, Saudi Arabia. As a tertiary care center, KFSH provides specialized services to a varied patient population. Its electronic health record system enabled efficient access to appropriate patient data, particularly for those receiving continuing care for diabetes. Patients involved in the study were adults aged 18 years or older who had an established diagnosis of Type 2 diabetes mellitus (T2DM), had at least one documented HbA1c result between August 2022 and July 2024, and attended at least one follow-up visit for diabetes management during the study period. Patients were excluded if they had significant comorbidities that could independently affect glycemic control, such as advanced chronic kidney disease (stage 4 or 5), liver cirrhosis, active malignancy, or congestive heart failure (NYHA (New York Heart Association) classes III-IV), or had incomplete or missing medical records (especially lacking HbA1c or follow-up data). The form used to extract data from the electronic medical records of eligible diabetic patients is shown in the Appendices.

Data source and data management

Patients’ electronic records at KFSH, from August 2022 to July 2024, were obtained. Following ADA (American Diabetes Association) recommendations for follow-up frequency, alongside typical clinical standards, patients were categorized into three groups based on the frequency of their follow-up visits. The first group comprised those with regular follow-up, defined as attending scheduled visits regularly, every three to six months throughout the study period. The second group contained patients with irregular follow-up, who had multiple visits but did not meet the recommended minimum number of visits. The third group consisted of patients with only a single recorded follow-up visit during the entire study period. A qualified family physician reviewed the records and extracted the data on age, gender, HbA1c levels, follow-up care frequency, lipid profile, and renal function parameters.

Laboratory parameters were classified based on standard clinical guidelines and the hospital’s laboratory reference ranges. HbA1c was reflected as controlled if it was less than 7%, and uncontrolled if it was 7% or higher, in agreement with the ADA guidelines. Triglyceride levels were reflected as high if they were 150 mg/dL or more, while low-density lipoprotein (LDL) cholesterol was reflected as high if it was 130 mg/dL or more. High-density lipoprotein (HDL) cholesterol was reflected as low if it was below 40 mg/dL for men or below 50 mg/dL for women. Total cholesterol was classified as high if it was 200 mg/dL or more.

Data analysis

Our descriptive statistics included means, standard deviations, frequencies, and percentages. To examine inferential tests, the chi-square test and Fisher's exact test were performed. Statistical significance was defined as a p-value of less than or equal to 0.05. SPSS software version 22.0 (IBM Corp., Armonk, NY, US) was used for data analysis.

Ethical considerations

The Regional Research Ethics Committee, Qassim, provided ethical approval for this study (Reg No: 607-46-6466). Utmost privacy and confidentiality were maintained for all data. Patient records were anonymized, and data access was limited to the research team. The collected data were securely stored in encrypted, password-protected files throughout the study and afterward. This ensured ethical research practices and adherence with data protection rules.

## Results

A total of 286 diabetic patients were included in the study. Of these, 175 (61.2%) were males and 111 (38.8%) were females, reflecting a male predominance in the study population. The majority were Saudi Nationals (n = 243, 85.0%), while 43 patients (15.0%) were non-Saudi.

Table 1 summarizes the clinical characteristics of the patients. The mean age of the patients was 60.5 (± 11.3) years. Additionally, the study population had an average BMI of 28.0 (± 5.2) kg/m2. The overall glycemic control was poor, as demonstrated by the mean HbA1c of 8.35%. With a mean LDL of 2.49 (± 1.01) mmol/L and a serum creatinine of 94.5 (± 75.3) umol/L, the lipid and renal profiles were within the normal ranges.

**Table 1:** Clinical characteristics of diabetic patients at King Fahd Specialist Hospital (August 2022 – July 2024) LDL: low-density lipoprotein; HDL: high-density lipoprotein

Variable	Mean ± SD	Range (Min–Max)	Valid N
Age (years)	60.5 ± 11.3	28 – 108	286
BMI (kg/m²)	28.0 ± 5.2	16.0 – 45.5	228
HbA1c (%)	8.35 ± 1.98	5.1 – 17.8	286
LDL (mmol/L)	2.49 ± 1.01	0.85 – 6.90	221
HDL (mmol/L)	1.02 ± 0.30	0.20 – 3.10	248
Triglycerides (mmol/L)	1.68 ± 1.08	0.30 – 8.50	226
Cholesterol (mmol/L)	4.00 ± 1.18	1.90 – 9.10	232
Serum creatinine (µmol/L)	94.5 ± 75.3	36 – 859	277

Table 2 summarizes the relationship between follow-up visit frequency and key metabolic markers. Glycemic control and follow-up status were found to be significantly associated (χ² = 15.892, p < 0.001), suggesting that patients who had regular follow-up had a higher chance of achieving good HbA1c levels. Total cholesterol (p = 0.100), triglycerides (p = 0.248), LDL cholesterol (p = 0.057), and HDL cholesterol (p = 0.118) did not show a statistically significant association with follow-up status; however, LDL cholesterol was almost significant.

**Table 2:** Association between follow-up visit frequency and metabolic markers among diabetic patients LDL: low-density lipoprotein; HDL: high-density lipoprotein

Follow-up Visits	Irregular	Single	Regular	χ²-value	p-value
Glycemic Control					
HbA1c <7%	14	40	17	15.892	<0.0001
HbA1c ≥7%	77	121	17		
Total Cholesterol					
Normal	75	120	27	3.997	0.1
High	1	6	3		
Triglyceride					
Normal	60	96	25	2.788	0.248
High	10	30	5		
LDL Cholesterol					
Normal	71	112	24	5.291	0.057
High	1	10	3		
HDL Cholesterol					
Normal/High HDL	54	64	18	4.22	0.118
Low HDL	32	67	13		

Table 3 details the relationship between glycemic control and lipid profile parameters. HbA1c levels and HDL cholesterol were found to be significantly associated (χ² = 5.249, p = 0.015), with low HDL more common in patients with inadequate glycemic control. Although triglycerides approached significance (p = 0.054), there were no statistically significant associations with total or LDL cholesterol.

**Table 3:** Association between glycemic control and lipid profile parameters among diabetic patients LDL: low-density lipoprotein; HDL: high-density lipoprotein

Lipid Profile	HbA1c <7%	HbA1c ≥7%	χ²-value	p-value
Total Cholesterol				
Normal	50	172	__	1
High	2	8		
Triglyceride				
Normal	50	131	3.949	0.054
High	6	39		
LDL Cholesterol				
Normal	54	153	__	0.526
High	2	12		
HDL Cholesterol				
Normal/High HDL	40	96	5.249	0.015
Low HDL	19	93		

**Figure 1:**
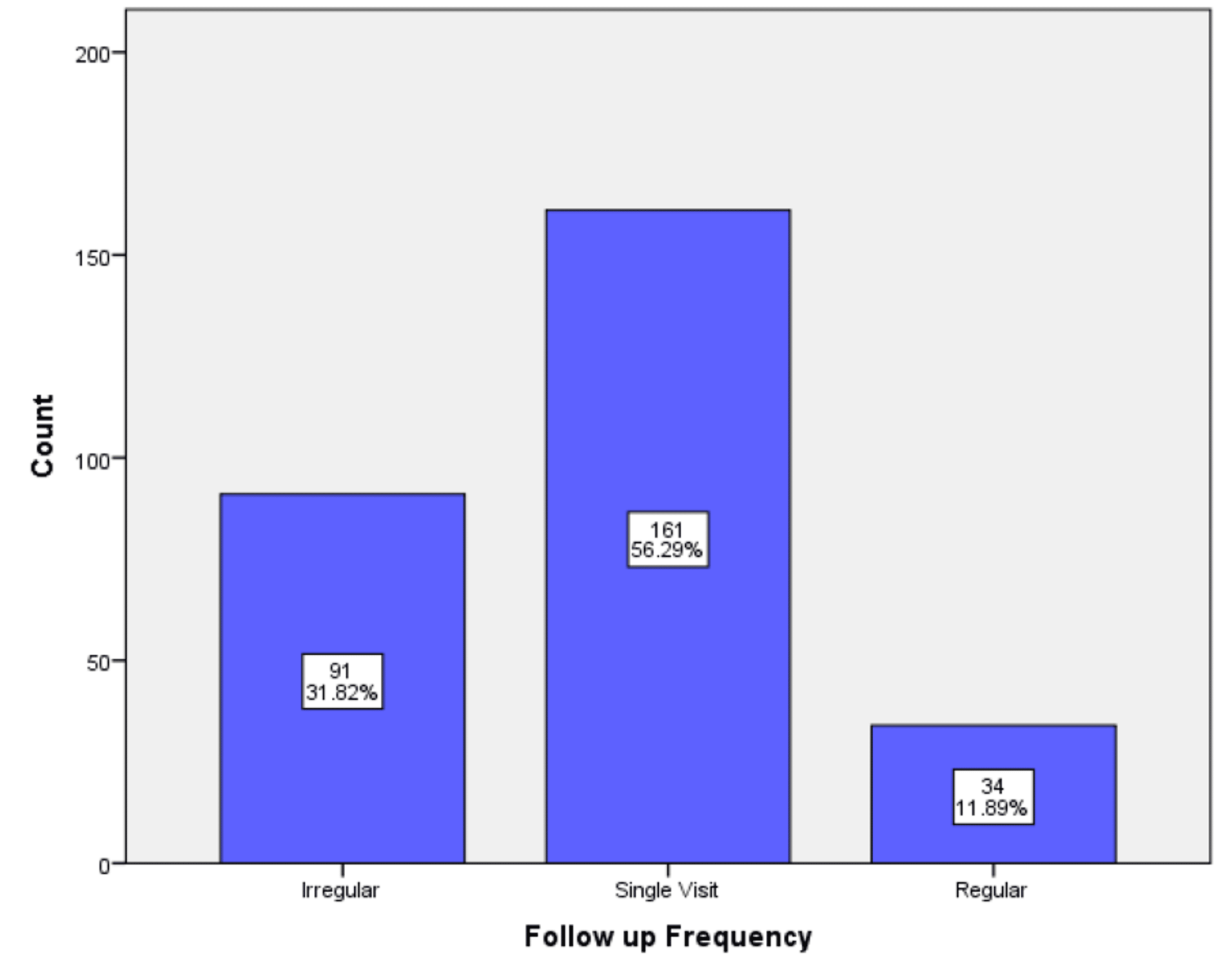
Distribution of diabetic patients by follow-up frequency

Figure 1 shows the distribution of patients by follow-up frequency. The majority of patients (56.3%) had only a single follow-up visit, while just 11.9% maintained regular follow-up, highlighting a gap in continuity of care among diabetic patients.

**Figure 2:**
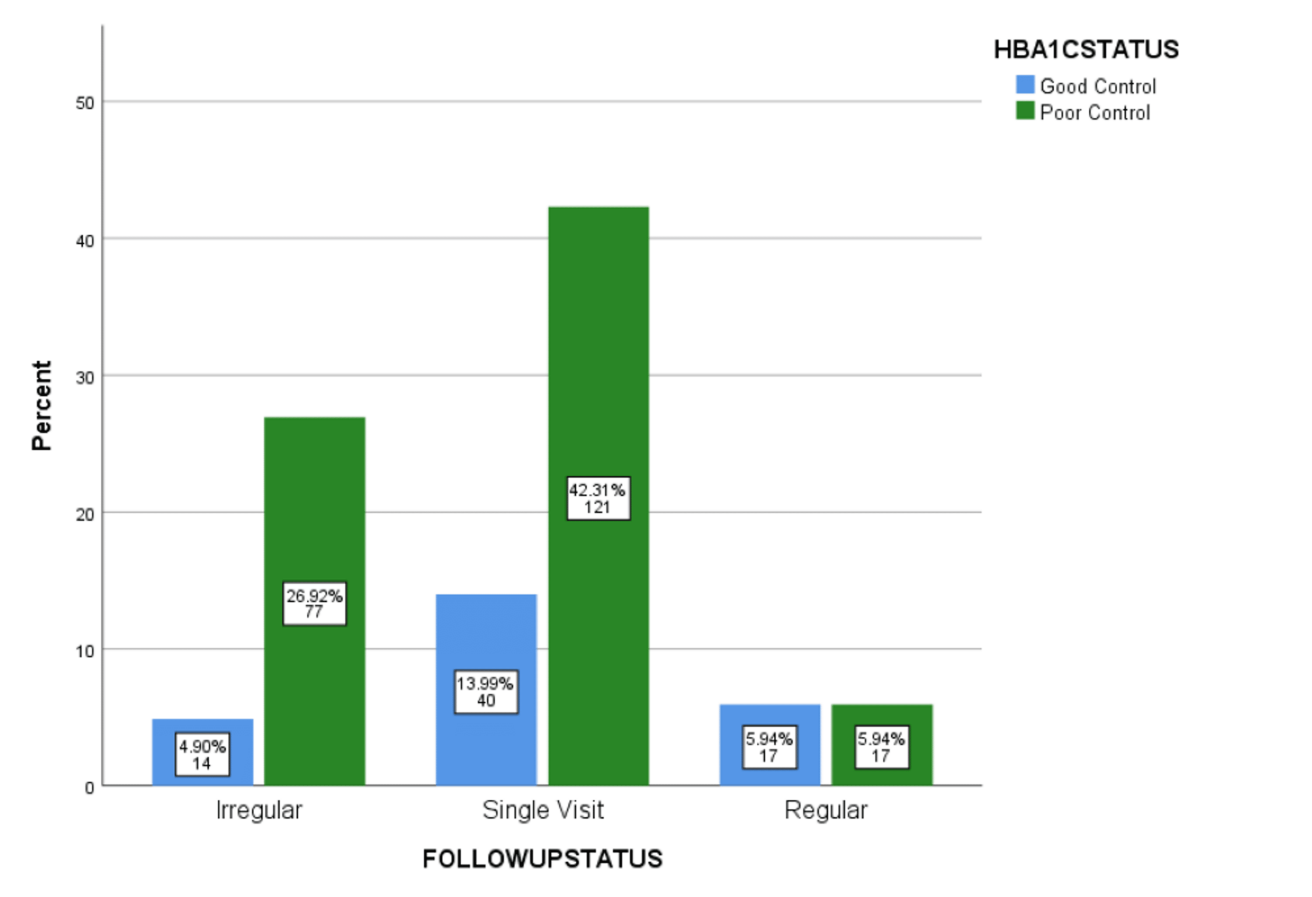
Glycemic control status of diabetic patients by follow-up frequency

Figure 2 shows the glycemic control status of diabetic patients by follow-up frequency. The percentage of regular visits was low for both groups (5.94%). However, individuals with poor glycaemic control had noticeably more irregular visits (26.92%) and single visits (42.31%) than patients with good control (4.90% irregular, 13.99% single). This shows clearly that individuals who do not receive regular follow-up typically have poorer blood sugar control.

**Figure 3:**
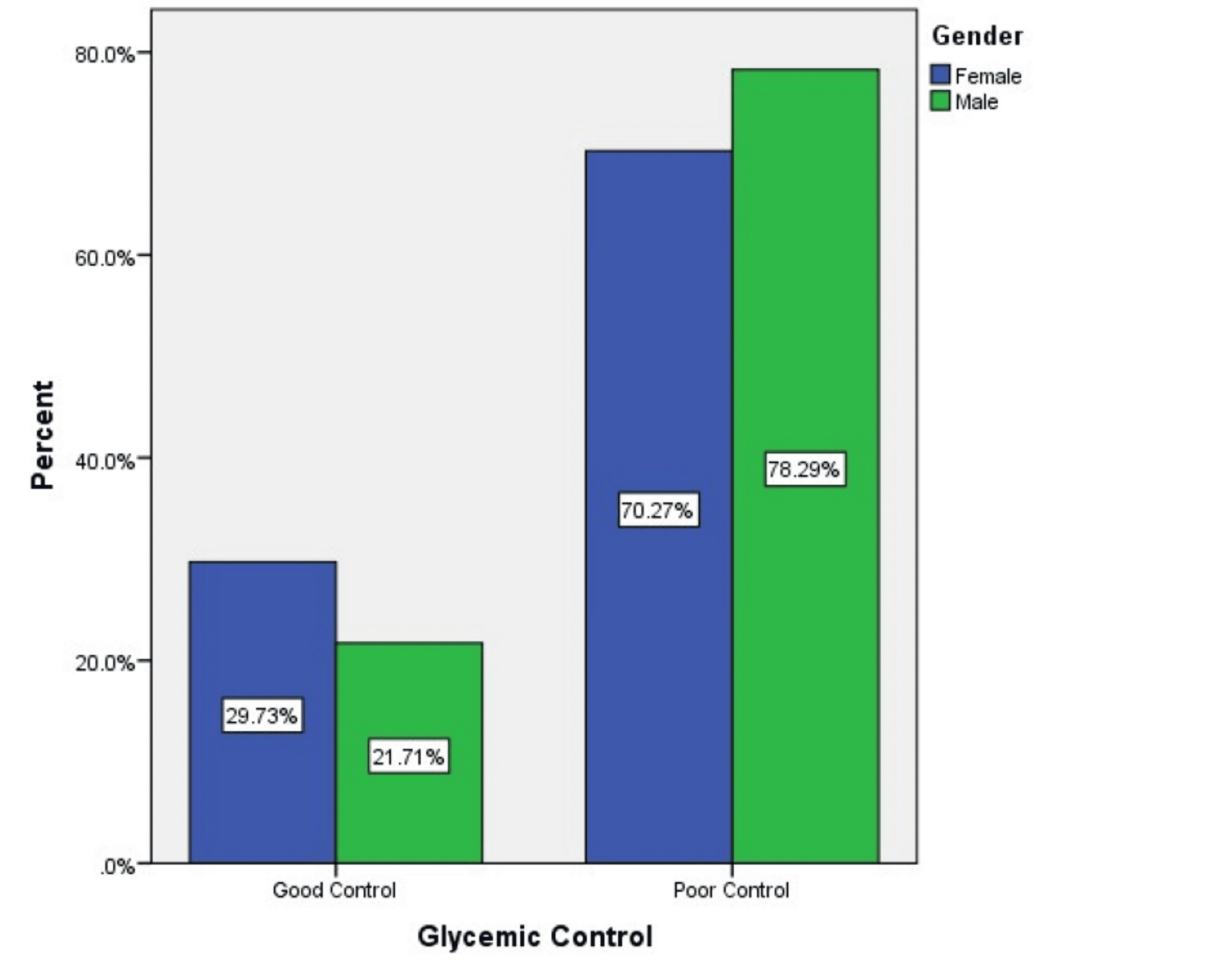
Distribution of glycemic control status by gender

Figure 3 illustrates glycemic control status by gender. A higher proportion of males (78.3%) had poor glycemic control compared to females (70.3%). Conversely, good control was more common among females (29.7%) than males (21.7%).

## Discussion

This study examined the association between glycemic control and the frequency of follow-up care among patients with diabetes in Qassim. Our findings showed that patients who had regular follow-up were significantly more likely to achieve good glycemic control (HbA1c < 7%) than those who only occasionally or infrequently obtained follow-up (χ² = 15.892, p < 0.001).

The ADA suggests routine HbA1c monitoring every three to six months and more frequently for those with poor control or treatment changes, emphasizing that continuity of care is a key determinant in successful diabetes management [11].

During the study period, more than half of the population (56.3%) had only one recorded follow-up, while only 11.9% had regular follow-up. The high rate of poor glycemic control observed (75.2% of patients with HbA1c ≥ 7%) may be partially explained by this, which highlights a significant gap in long-term care continuity. These findings are consistent with previous studies conducted in Saudi Arabia, which reported the same patterns in limited follow-up adherence and its link to suboptimal outcomes [12,13]. In addition, nearly three-quarters of patients with type 2 diabetes in northern Saudi Arabia had poor glycemic control, with contributing factors including obesity, long disease duration, and poor adherence to lifestyle measures [14].

Low HDL cholesterol was linked to poor glycemic control (χ² = 5.249, p = 0.015), supporting evidence that HDL is a marker of cardiovascular protection and insulin sensitivity [15]. Although no significant associations were found between LDL and triglyceride levels, their borderline p-values (p = 0.057 and 0.054, respectively) suggest that correlations may become more apparent in a larger sample or over a longer study period.

The metabolic and clinical profile of this study, with a mean BMI of 28.0 kg/m², elevated HbA1c, and modest lipid abnormalities, is consistent with the typical cardiometabolic risk cluster seen in patients with T2DM. These findings highlight the importance of integrated care models that not only monitor glucose but also address weight, blood pressure, and lipid control during routine follow-up, as emphasized in current international guidelines and demonstrated in local interventional research [11,16]. Moreover, Tourkmani et al. revealed that applying a structured, multidisciplinary care program with regular follow-up led to significant improvements in HbA1c, lipid profiles, and blood pressure control [16].

Despite the value of using real-world clinical data, this study has limitations. Sociodemographic factors that are known to affect the course of chronic diseases, such as access to care, education, and income, were not evaluated. Based on the results of our study, we recommend increasing awareness about the sustained benefits of regular follow-up of diabetic patients in healthcare settings. Further research is recommended to investigate the relationships between specific follow-up schedules and long-term metabolic results.

## Conclusions

This study showed a statistically significant relationship between glycemic control and frequency of subsequent testing in diabetic patients in Qassim. Individuals who consistently attended their scheduled follow-up visits demonstrated a significantly increased likelihood of achieving their HbA1c targets. This highlights the positive impact of routine, scheduled appointments in improving health outcomes and decreasing the incidence of diabetes-related complications.

## Appendices

**Table 4:** Data extraction form This form was used to extract data from the electronic medical records of eligible diabetic patients during the study period from August 2022 to July 2024. LDL: low-density lipoprotein; HDL: high-density lipoprotein; AST: aspartate transferase; ALT: alanine aminotransferase

Variable	Value
Patient ID (anonymized)	
Age	
Gender	
Nationality	
HBA1C at Each Visit	
Dates of all Included Visits	
Number of Follow-Up Visits	
Duration Between Each Visit	
Frequency of Follow-Up	
LDL	
HDL	
Total Cholesterol	
Triglycerides (TG)	
AST	
ALT	
Creatinine	
Blood Pressure	
BMI	
